# An Update on the Use of Lasers in Prosthodontics

**DOI:** 10.7759/cureus.57282

**Published:** 2024-03-30

**Authors:** Abdulaziz Binrayes

**Affiliations:** 1 Department of Prosthetic Dental Science, College of Dentistry, King Saud University, Riyadh, SAU; 2 Department of Dentistry, Vision College, Riyadh, SAU

**Keywords:** soft tissue laser, dental implants, fixed prosthodontics, prosthodontics, laser

## Abstract

Lasers are employed in all fields of modern dentistry nowadays, including both surgical and non-surgical dental procedures. Prosthodontics, a branch of dentistry, has also embraced lasers as an invaluable addition to conventional methodologies. This helps improve the standard of care for patients and dentists due to its precise excision, quick healing, and enhanced tissue response after surgeries. In prosthodontics, the most commonly used lasers are carbon dioxide, argon, and yttrium-aluminum-garnet (YAG) lasers. Many reviews have been published in the literature regarding the use of lasers in dentistry; however, reviews on the use of lasers in the field of prosthodontics are limited. This review aims to explain the diverse applications and advancements of lasers in prosthodontics. Furthermore, it will highlight the integration of lasers in diagnostic protocols, treatment modalities, and the fabrication of prosthetic restorations.

## Introduction and background

Lasers are employed in all fields of modern dentistry, including conservative dentistry, endodontics, periodontology, implantology, oral surgery, and other dental procedures [1]. Prosthodontics, one of the main branches of dentistry, has embraced lasers as an invaluable addition to conventional methodologies. Lasers provide a new standard of care and application in both fixed and removable dental prostheses. This helps improve the standard of care for patients and dentists due to its precise excision, quick healing, and enhanced tissue response after surgeries [2].

Lasers have been employed in both surgical and non-surgical procedures in prosthetic dentistry. They are used for the treatment of periodontitis and the successful management of peri-implant lesions. Furthermore, aesthetic dentistry has utilized lasers for the removal of tooth decay debris, reducing recrudescent decay, and helping in bleaching. For oral tissue the commonly used lasers are carbon dioxide and Nd:YAG lasers [3]. For oral tissue, the commonly used lasers are carbon dioxide and Nd:YAG lasers [3]. For soft tissue management, diode lasers are mostly utilized. The common applications for soft tissues with lasers include gingival reshaping and troughing, gingivectomies, frenectomy, incisions of the soft tissues, and removal of mucosal lesions of the oral mucosa, including leukoplakia, lichen planus, etc. Additionally, laser use in surgical procedures can reduce scarring, maintain hemostasis, decrease post-operative pain, edema, and inflammation of the affected tissue. Furthermore, it can help in treating microbial infections in the oral cavity and assist in rehabilitation after major procedures [4]. Recently, it has also been reported that laser irradiation may reduce root resorption and favor periodontal regeneration following the replantation of avulsed teeth [5].

Lasers stand for the acronym 'Light Amplification by Stimulated Emission of Radiation.' In the field of clinical dentistry, they have been in use since the 1960s [3,4]. However, major breakthroughs in prosthodontics occurred in the 1990s. The US FDA (fda.gov) approved a pulsed Nd:YAG laser for intraoral soft tissue use [4]. Subsequently, lasers of different wavelengths such as Argon, Ho:YAG, and Er:YAG were investigated for dental practice [6]. Different lasers are categorized based on their wavelengths, such as the diode laser with a 810-890 nm wavelength; Nd:YAG at 1064 nm; CT:YSGG at 2780 nm; Er:YAG at 2940 nm; and CO₂ lasers with a wavelength of 10600 nm [7].

There have been many reviews written about the use of lasers in dentistry, but not as many focusing on their application in prosthodontics. This review's objectives are to assess, condense, and clarify the many different applications and developments of lasers in clinical prosthodontics. The purpose of this review is to elucidate the various uses and advances of lasers in prosthodontics. Additionally, it will illustrate how lasers are integrated into diverse prosthodontic treatment modalities and diagnostic techniques. The many classes of lasers and how they interact with oral tissue will also be highlighted in the review. Furthermore, it will provide a summary of the clinical applications, risks, safety measures, and potential applications in prosthetic dentistry research. Overall, this review is anticipated to serve as a guide for innovative laser applications in prosthodontics, with the goal of advancing clinical practice.

## Review

Different types of lasers used in prosthodontics

Many types of lasers are used in dental practice. The most commonly used are:

Carbon Dioxide (CO₂) Lasers

These CO₂ lasers primarily operate in the infrared spectrum with a wavelength of 10,600 nm. They are primarily used for soft tissue procedures in prosthodontics. The benefits of CO₂ lasers include their strong absorption by water-containing tissues, making them suitable for precise ablation of soft tissue, such as gingivectomies, frenectomy, excisional biopsy, and crown lengthening. They can also be used for depigmentation. Furthermore, they can easily cut and coagulate soft tissue and have a shallow depth of penetration [8]. More advanced, a 9,300-nm wavelength CO₂ laser is used for dental hard tissue removal, making CO₂ lasers increasingly useful for dental hard tissue preparation [3].

Diode Lasers

Diode lasers mainly operate in the visible or near-infrared spectrum, with wavelengths ranging from 810-980 nm. They are used in both contact and non-contact modes and applied in both continuous wave and gated pulse modes in dentistry [9]. Diode lasers are versatile, with applications in both soft tissue management and certain restorative procedures. They are known for advantages such as decreased cellular destruction, minimal bleeding and tissue swelling, enhanced visualization of the surgical area, minimal scarring with minimal or no suturing requirement, and less post-surgical pain. Diode lasers offer numerous benefits due to their small size, easy operability, and cost-effectiveness. They are mostly used in gingivoplasty, gingivectomy, implant recovery, laser soft tissue curettage, soft tissue crown lengthening, and sulcular debridement, among others [9].

Erbium Family Lasers (Er:YAG and Er,Cr:YSGG)

Erbium lasers operate at higher wavelengths in the range of 2780 nm (erbium-chromium-doped yttrium-scandium-gallium-garnet (Er,Cr:YSGG)) and 2940 nm (erbium-doped yttrium-aluminum-garnet (Er:YAG)). They are most effective in the management of hard tissue applications as they primarily target tissue water and have a high affinity for hydroxyapatite. They are known for eliminating the smell and vibration associated with dental handpieces [10-11]. They are used for precise and conservative ablation of dental hard tissues, such as cavity preparation and enameloplasty, while minimizing thermal damage.

Mechanism of action and interaction with oral tissues

Absorption and Tissue Interaction

Lasers used in prosthodontics interact with oral tissues through different mechanisms. For instance, CO₂ lasers' energy is absorbed by water and on the surface of the oral soft tissue [12]. The visible part of the spectrum (445 nm-660 nm) is mainly absorbed within the first centimeter of soft tissue, primarily by pigmented chromophores including melanin and hemoglobin. Since the Erbium wavelength corresponds with the absorption peak of water and hydroxyapatite, it allows the elimination of calcified dental substance through micro-explosions, making Er:YAG therapy a regularly utilized technology for hard dental tissue ablation [13].

Tissue Effects

Laser energy is predominantly delivered in the form of repeated pulses or sometimes as continuous waves. This leads to various effects on the tissues, such as soft tissue lasers primarily leading to the vaporization or ablation of the damaged area [14]. Meanwhile, hard tissue lasers, such as the erbium laser, induce very precise removal of the extra or unwanted debris through photothermal or photomechanical effects [15].

Selective Photothermolysis

The principle of selective photothermolysis involves the delivery of specific laser light wavelengths for precise and selective tissue interaction. Thus, it targets a particular chromophore only, without affecting any of the surrounding tissues. This unique property enables selective tissue ablation or coagulation, which is very crucial in precise prosthodontic procedures such as crown remodeling, tooth implantation, and other prosthesis replacements in the oral cavity [16].

Laser parameters and their significance in prosthodontics

Laser Wavelength

Different laser parameters have different significance. For example, different wavelengths of lasers have varying affinities for soft and hard oral tissues. It is of extreme importance to understand the optimal wavelength required for a particular procedure. This is crucial to achieving optimal tissue interaction in order to minimize collateral damage and reduce the complications of the procedure [17].

Laser Pulse Duration and Energy Density

Secondly, the optimum control of pulse duration and energy density of different laser pulses is essential for optimizing the tissue effects. Shorter pulses minimize heat diffusion and reduce thermal damage to adjacent tissues [18].

Spot Size and Delivery Systems

During procedures, precision and control are affected by the laser beam's spot size as well as the type of delivery system (handpieces, fibers). When all these variables of the delivery system are adjusted appropriately, the prosthodontic procedures may produce very efficient results [16].

Clinical applications

Soft Tissue Management

Lasers have been shown to be incredibly useful in a variety of procedures, and the management of soft tissue is an integral part of prosthodontic therapies. For example, in gingivectomy and gingivoplasty, where there is removal and reshaping of the gingival tissue, different lasers are routinely used due to their exact precision and minimal invasiveness to the surrounding tissue [19]. Laser-assisted procedures offer many advantages, such as reduction of bleeding, controlled tissue ablation, and improvement in patient comfort and satisfaction during and after the intervention [20].

In addition, lasers have proven to be very effective in crown lengthening and resizing procedures that aid in the adequate exposure of tooth structure for various restorative purposes. This ability to precisely remove soft tissue without causing injury to adjacent structures adds to the success of such procedures [21]. Furthermore, frenectomy and frenotomy procedures, aimed at the correction of aberrant muscle attachments in the oral cavity, also benefit from laser precision, leading to enhanced healing and reduction of postoperative discomfort for patients [22]. The use of lasers in soft tissue management within prosthetic procedures not only ensures better clinical outcomes but also promotes patient satisfaction by decreasing procedural discomfort and accelerating recovery.

Gingivectomy and Gingivoplasty

Many lasers, particularly diode and CO₂ lasers, are employed for reshaping, resizing, and contouring the gingival tissues. These procedures aid in augmenting the appearance of the gingiva, adjusting gingival levels, and preparing the soft tissue architecture for esthetic purposes and prosthetic restorations [19].

Crown Lengthening

Laser-assisted crown lengthening involves the recontouring of gum tissue and bone to expose more tooth structure. This procedure is commonly performed before restorative treatments to ensure there is adequate tooth structure for crown placement [21].

Frenectomy and Frenotomy

Lasers are used to remove or modify frenula, such as lingual and labial frenula, to alleviate issues like restricted movement, speech impediments, or for addressing removable prosthodontics limitations, such as high frenulum attachments causing soreness and irritation in complete dentures [22].

Implant dentistry

Soft Tissue Contouring Around Implants

Laser-assisted techniques help in shaping and contouring peri-implant soft tissues to achieve better esthetic outcomes and create natural-looking emergence profiles around dental implants [23].

Decontamination of Implant Surfaces

Lasers, particularly erbium lasers, are utilized for peri-implantitis management by debriding and disinfecting implant surfaces without causing damage to the implant or adjacent tissues [23].

Peri-implantitis Management

Laser therapy aids in the treatment of peri-implantitis by reducing inflammation, decontaminating infected tissues, and promoting better tissue attachment, thus enhancing the long-term success of implant-supported prostheses [24].

Restorative procedures

Cavity Preparation and Caries Removal

Erbium lasers are used for precise and conservative removal of carious tissues, preserving healthier tooth structure compared to traditional drilling methods. Many of these offer minimal discomfort during dental procedure and often eliminate even the need for local anesthesia [25].

Bonding and Cementation Protocols

Laser engraving and conditioning of the tooth surface and intaglio surface of the restorative materials, improves adhesive bonding in various restorative materials used in prosthodontics. This enhances the longevity and strength of the prosthetic restorations [15].

Ablation of Dental Materials

Lasers also facilitate the efficient removal of old and worn out restorative materials, which include composites or amalgams etc. Minimizing the damage to the surrounding hard and soft structure of the oral cavity [16].

Adjunctive therapies

Photo-biomodulation (Low Level Laser Therapy)

The Laser therapy is also employed in order to stimulate healing process, reduce inflammation of the tissues, and alleviate the pain in post-prosthodontic treatments. This also promoting a faster recovery and enhances patient comfort [26].

Laser-Assisted Periodontal Therapy in Prosthetic Cases

Lasers also aid in different periodontal health maintenance around the newly added prosthetic restorations. This is assisting in managing different conditions like peri-implant mucositis, infections and gingival inflammation [26].

Advantages and limitations

Advantages of Laser Applications in Prosthodontics

Precision and minimally invasive approach: Lasers offer exceptional precision in tissue ablation and tooth preparation for prosthetic procedures. They allow for conservative treatment methods by selectively targeting specific tissues without affecting surrounding parts [12]. This high precision is particularly beneficial in dental prosthetics, where intricate work is frequently required for restorations and soft tissue management.

Reduced discomfort and faster healing: Compared to traditional methods, laser-assisted procedures typically result in less post-operative discomfort for patients. Lasers promote faster tissue healing by sealing nerve endings, reducing bleeding, and minimizing trauma to surrounding tissues, contributing to a more comfortable patient experience [26].

Hemostatic properties and bacterial decontamination: Certain types of lasers possess hemostatic properties, enabling blood clot formation during soft tissue procedures. Additionally, lasers can aid in microbial decontamination, reducing the risk of bacterial infections and improving the success rates of prosthodontic treatments [27].

Versatility in applications: Laser assisted procedure find diverse applications in prosthodontics, that ranges from soft tissue management (for example, gingivectomy, crown lengthening) to several restorative procedures (for example, cavity preparation, bonding), and even in the implant dentistry including the peri-implantitis management, soft tissue contouring etc [15,21].

Enhanced patient experience: The reduction in the need for anesthesia, minimal discomfort during and after procedures, and quicker recovery times all contribute to an overall improved patient experience. This leads to higher patient satisfaction rates in clinical practice.

Limitations of laser applications in prosthodontics

Cost Considerations and Equipment Investment

It is of special consideration that acquiring laser technology for clinical uses and maintaining these devices could be financially very demanding for dental practices and clinics. The initial investment of the equipment, maintenance, and periodic servicing of these laser equipment can pose a substantial financial burden.

Operator Training and Learning Curve

The proper utilization of laser technology in prosthodontics requires a specialized training and expertise in order to obtain optimum results. Dentists who are interested in lasers must undergo a comprehensive training to understand the nuances of lasers. They should have a sound knowledge of different laser types, appropriate parameters for use, and safety protocols. The learning curve can be steep at times, requiring time and dedication [1].

Restricted Access in Certain Anatomical Areas

Some laser systems used in dentistry may have limitations in accessing certain areas of the oral cavity due to their design or wavelength characteristics. These limitations can pose challenges while performing procedures in challenging-to-reach locations [28].

Tissue Interaction and Thermal Damage

Improper usage or incorrect settings protocols of lasers may lead to thermal damage of the surrounding tissues. Furthermore, different tissues respond in diverse fashion to laser energy. Interaction between the laser and tissue can sometimes lead to unexpected and unpredictable outcomes [12].

Limited Evidence-Based Research for Certain Applications

While lasers have shown promising results in various procedures, there might be a lack of robust evidence for certain applications. Long-term clinical studies and evidence-based research are lacking to support their efficacy in certain applications, leading to uncertainties regarding long-term success and outcomes in many laser-assisted treatments.

Patient outcomes and safety considerations

Laser-assisted Procedures and Patient Experience

Enhanced comfort and reduced discomfort: Laser-assisted procedures in prosthodontics often result in reduced discomfort during and after treatment compared to traditional methods. Patients commonly experience less pain, decreased bleeding, and reduced swelling in the oral cavity, leading to improved comfort during the recovery period [29].

Faster healing and recovery: Laser promotes accelerated tissue healing in all procedures due to its precise tissue interaction, reduced trauma to tissues, and minimal thermal damage, leading to quicker recovery times for patients undergoing any laser-assisted prosthodontic procedure.

Improved esthetic outcomes: The precision in soft tissue management with lasers enhances esthetic outcomes in many procedures, such as gingival contouring and crown lengthening, resulting in improved smiles and aesthetically pleasing outcomes for all patients [29].

Long-term success of prosthetic restorations: Procedures with laser-assisted technology contribute to better integration of prosthetic restorations. This also leads to improved bonding and higher longevity of dental materials. Laser helps in ensuring the long-term success of treatments [16].

Safety guidelines and precautions of lasers

Safety Considerations

Eye protection: It is very important to have adequate eye protection for both the patient and dental team during any laser procedures. Properly designed protective eyewear that filters out specific wavelengths must be worn throughout the procedure to prevent potential ocular damage [30,31].

Tissue heating and thermal damage: Careful control of laser parameters, such as power settings and exposure times, is essential to avoid excessive tissue heating and thermal damage. Continuous water spray or air-cooling methods are often used to mitigate thermal effects during laser procedures [31].

Patient evaluation and selection: This is highly important to consider before lasers. Sometimes not all of the patients may be suitable candidates for laser-assisted treatments. Proper patient evaluation must be done which includes medical history and assessment of oral tissues. This is necessary in order to determine the feasibility and safety of laser applications.

Operator training and competence: The dentists or prosthodontists performing laser procedures must undergo a proper comprehensive training and possess necessary expertise in order to handle laser equipment effectively. In addition, adequate knowledge of the laser physics, its tissue interaction, and all safety protocols is imperative to ensure safe and efficient treatments.

Infection control measures: Aseptic techniques with strict adherence to the infection control protocols are necessary. This includes proper sterilization of laser tips or hand pieces or any instruments coming in contact and maintaining aseptic conditions during all procedures. It is crucial to prevent any potential cross-contamination or infections [27].

Long-term outcomes and follow-up considerations

Postoperative Care and Follow-up

Providing patients with appropriate postoperative care instructions and scheduling follow-up appointments to monitor tissue healing and address any complications are essential steps to ensure optimal patient outcomes and safety.

Future implications and emerging trends

Technological Advancements in Laser Systems

Improved laser delivery systems: With the passage of time, advancements in laser delivery mechanisms, such as more ergonomic handpieces and flexible fiber optic delivery systems, may enhance precision and accessibility in various prosthodontic procedures.

Wavelength specificity and customization: Different laser systems are evolving to offer a greater wavelength specificity. This will help in allowing for more tailored treatments based on different tissue types and specific treatment objectives. Customizable laser parameters will enable more accurate tissue interactions [7].

Potential Integration of Lasers With Digital Dentistry

Integration with digital dentistry: The amalgamation of lasers with digital imaging and different CAD/CAM technologies is a burgeoning trend in research and clinical practice. Laser scanning of the affected area combined with digital impressions will facilitate more detailed treatment planning and design for prosthetic restorations [32].

Expansion of Applications in Prosthodontics

Regenerative therapies: Lasers have shown promising results in promoting tissue regeneration and enhancing the osseo-integration around dental implants. Research also continues to explore other potential of lasers in many regenerative techniques for better outcomes in prosthetic treatments [20].

Nanotechnology and biomaterials: Combining laser technology with nanotechnology is leading to the development of novel biomaterials and new techniques for surface modifications, aiming to improve the biocompatibility, bioavailability, and durability of dental implants and restorative materials [33].

Pain management and neuromodulation: It is an emerging field that will help in the exploration of low-level laser therapy (LLLT) for managing postoperative pain and reducing inflammation. This might be due to the possible influence of nerve regeneration around prosthetic restorations which is an emerging area of interest for many researchers and clinicians.

Unexplored Avenues and Research Directions in Laser-based Prosthodontics

Bio-stimulation and tissue engineering: The use of laser energy to promote tissue regeneration and repair is the aim of bio-stimulation. It is a feasible treatment for the regeneration of both soft and hard tissues. Research has demonstrated that it significantly enhances the osseointegration and biomaterials' characteristics. The use of lasers in bone regeneration is still not covered by an established protocol of work that has been approved, thus research in this area is necessary since it could produce encouraging results. Utilizing lasers in tissue engineering techniques could transform the development of new biocompatible materials for prosthodontic applications [34].

Artificial intelligence (AI) integration: AI-driven technologies may assist in treatment planning, precision, and predictive analysis in laser-assisted prosthodontic procedures, optimizing laser settings for personalized treatment protocols [30].

Translational research and clinical trials: Continued translational research and well-designed clinical trials are essential to validate the efficacy, safety, and long-term outcomes of emerging laser-based interventions in prosthodontics [32-34].

Environmental and Cost Considerations

Energy-efficient laser systems: The development of more energy-efficient laser systems that minimize power consumption and waste heat is an area of interest, addressing environmental concerns and cost-effectiveness [35].

Affordability and accessibility: Efforts to make laser technology more affordable and accessible to a broader range of dental practices may democratize its use in prosthodontics, potentially benefiting a larger patient population [36].

## Conclusions

In every area of dentistry, lasers are regarded as extremely helpful instruments. In the near future, lasers may be preferred over traditional surgical and therapeutic methods due to technological advancements. The use of laser therapy in prosthodontics, a cutting-edge instrument, is expected to lead to faster treatment and healing outcomes, producing predictable clinical results. A laser facilitates a quicker and easier procedure with little to no discomfort, causing less tissue damage. However, a suitable learning curve is necessary when using any kind of laser to maximize therapeutic benefits and prevent adverse effects. The combination of therapeutic and diagnostic laser techniques represents another area of future expansion. Looking ahead, specific laser technologies are anticipated to become indispensable to modern dentistry practices, significantly improving dental care.
